# Electrophoretic profile of seminal proteins and their correlation with *in vitro* sperm characters in Black Bengal buck semen

**DOI:** 10.14202/vetworld.2019.621-628

**Published:** 2019-05-03

**Authors:** M. Karunakaran, Vivek C. Gajare, Ajoy Mandal, Mohan Mondal, S. K. Das, M. K. Ghosh, S. Rai, R. Behera

**Affiliations:** ICAR-National Dairy Research Institute, Eastern Regional Station, Kalyani, West Bengal, India

**Keywords:** buck, *in vitro* characters, semen, seminal proteins

## Abstract

**Aim:**

This study aimed to study the electrophoretic properties of seminal plasma and sperm proteins of Black Bengal buck semen and their correlation with *in vitro* sperm characters and freezability.

**Materials and Methods:**

Semen ejaculates from nine Black Bengal bucks were collected by artificial vagina (n=20/buck). Ejaculates were evaluated for *in vitro* sperm characters and electrophoretic profile of seminal protein. *In vitro* sperm characters were evaluated immediately after collection, after completion of equilibration period, and after freeze-thawing. For seminal protein studies, seminal plasma proteins were precipitated by ice-cold ethanol method, and sperm proteins were extracted by Triton X detergent extraction method. Discontinuous sodium dodecyl sulfate-polyacrylamide gel electrophoresis (SDS-PAGE) was performed to assess the molecular weight of seminal proteins. Correlation between *in vitro* sperm characters and protein bands was determined by Pearson’s correlation coefficient, and two-way ANOVA was applied to find the individual buck differences.

**Results:**

Significant difference (p<0.01) among the bucks was noticed in the *in vitro* sperm characters evaluated at all the three stages of semen evaluation such as immediately after collection, after completion of equilibration period, and post-freeze thawing. Progressive loss of sperm motility, membrane integrity, and other *in vitro* sperm characters were noticed during cryopreservation. A total of ten protein bands in the molecular weight ranging from 17 to 180 kDa were found in the SDS-PAGE of seminal plasma proteins, while nine bands of 17-134 kDa were observed in sperm proteins. Seminal plasma proteins of molecular weight 75, 62-49, 20, and 17 kDa and sperm proteins of 75, 20, and 17 kDa were present in all the nine bucks (100%) screened, and variation among the bucks was noticed for the presence of other proteins. Seminal plasma protein of 180-134 kDa showed a negative correlation with individual motility (−0.716) and functional membrane integrity of sperm cells (−0.724) in post-freeze–thaw analysis and 48 kDa protein had a positive correlation with individual motility (0.649) and functional membrane integrity of sperm cells (0.664) in post-thaw analysis. Sperm proteins of 63 kDa had a negative correlation (−0.616) with sperm concentration in neat semen.

**Conclusion:**

Variation among the bucks was noticed in the *in vitro* sperm characters and semen freezability. Correlation between seminal proteins and *in vitro* sperm characters and semen freezability had been found which might be useful as a tool to select breeding bucks.

## Introduction

Animal husbandry and dairying are integral parts of human life since the process of civilization. Bengal goat is known for its meat and skin quality, adaptability, and high fecundity. It attains sexual maturity at an early age, and the female goat becomes pregnant twice a year and gives birth to 1-3 kids [1]. Black Bengal breed goats are found in West Bengal, Bihar, Jharkhand, Odisha, North Eastern India, and neighboring country Bangladesh. Most of the goat keepers are small and marginal farmers, landless laborers having a flock size of 3-5 animals. Although the ratio of male and female kids at birth was 47.9:52.9, the ratio of sexually matured buck and doe was reduced to 1.13:88.7. This was mainly due to early castration and sale of the male goats at 9-12 months of age for meat purpose [2]. It leads to less availability of breeding bucks in the field, and the does are bred indiscriminately with available males and result in dilution/loss of valuable germplasm [3]. Artificial insemination (AI) technology has made possible the safe use of semen from selected sires in a large breeding female population. AI in goat is gaining popularity in several states of India such as Tamil Nadu, Kerala, West Bengal, Assam, and others for the past few years [4-6]. AI costs less when compared to keeping breeding buck(s) in small flocks of 3-5 goats, and the farmer has access to a wide variety of quality bucks of high genetic merit at relatively low cost.

While adapting AI technology, accurate evaluation of male fertility is important because it influences the reproductive potential of a large population of females. Currently, breeding soundness examination (BSE) is carried out before introducing a male into the semen collection program. The variations in the fertility rate among the males which had passed through BSE were not addressed by the routine semen evaluation parameters [7]. Attention is now being directed toward the assessment of other aspects of semen quality as predictors of fertility. Proteins present in the seminal plasma and sperm have been reported as markers of fertility [8-10].

Seminal plasma, a complex mixture of secretions from the testis, epididymis, and accessory sex glands, contained factors that modulated the fertilizing ability of sperm [11]. The role of seminal plasma proteins in the regulation of sperm function was highly complex and was manifested during different molecular events. Several studies provided direct evidence that proteins of seminal plasma were adsorbed to the surface of sperm [12] and affected its function and properties [13]. Some of these proteins were probably adsorbed onto the surface so tightly that they would be inseparable or indistinguishable from the “intrinsic” membrane proteins, while others may be removed by simple washing [14]. The proteins were topographically reorganized into specific regions of the sperm surface and changed the properties of the sperm membrane by binding to it and/or modifying the structure or the arrangement of the existing membrane molecules. It was suggested that these proteins maintained the stability of membrane up to the process of capacitation. Capacitation and subsequent acrosome reaction reduced the content of proteins on the sperm surface, and approximately 35% of them only remained on the spermatozoa after acrosomal reaction [15]. Studies on the proteome of accessory sex gland fluid from sires of high and low fertility revealed the overexpression of seminal vesicle proteins, such as spermadhesins in low fertility sires and osteopontin in high fertility sires. Both proteins are produced in the seminal vesicles, and these proteins interact with the sperm membrane during capacitation and assist penetration of the oocyte [16]. Proteins such as osteopontin, prostaglandin-D synthase, bovine seminal plasma proteins (A1, A2, and A3), and heparin-binding proteins (HBPs) have been reported as indicators of bull fertility [16-18]. Although many works have been carried out on seminal proteins in bovine, equine, and other species, only a few studies have been carried out on buck semen.

Accumulating evidence has indicated that inherent male variability in semen freezability is one of the factors responsible for marked differences in the sperm cryosurvival [19,20]. Studies have reported that differences in sperm freezability might be due to a genetic origin [21]. Even though the underlying mechanisms responsible for the genetic differences associated with poor or good semen freezability are yet unknown, it has been suggested that the identification of sperm freezability markers might be the most efficient approach to improve the technology of semen cryopreservation. Considering the present demand and future scope of preserved goat semen, it is essential to identify markers for the selection of breeding bucks for semen donation.

This study aimed to study the *in vitro* sperm characters during different stages of cryopreservation, to isolate and characterize seminal plasma and sperm proteins, and to find the correlation between seminal proteins and *in vitro* sperm characters and semen freezability in Black Bengal buck semen.

## Materials and Methods

### Ethical approval

The experiment was carried out with the approval of the Institute Research Council of ICAR-National Dairy Research Institute (ICAR-NDRI), Karnal. Semen from the bucks was collected using artificial vagina as per the standard practice of semen collection from livestock species.

### Experimental animals

The present study was carried out at ICAR-NDRI, Eastern Regional Station (ERS), Kalyani, West Bengal, India. Kalyani is situated at 22° 58’30”N latitude and 88° 26’ 4” E longitude. The climatic condition is hot humid. Nine Black Bengal bucks (*Capra hircus*) were used in the study. Bucks were given with the identification tag numbers such as 46, 48, 51, 52, 53, 55, 57, 59, and 67. All the experimental animals were clinically normal and donating semen of acceptable quality. Semen ejaculates were collected twice a week using artificial vagina. Before semen collection, bucks were sexually prepared by allowing 1-2 false mounts [4,5]. On every collection day, two ejaculates were collected from each buck with a brief interval. These two successive ejaculates from each buck were mixed; half was used for *in vitro* studies and another half was used for protein studies. A total of 20 ejaculates from each buck were used in the study. Semen ejaculates were collected during the period of November 2017-February 2018. Semen ejaculates with 3+ and above mass motility and 70% and above individual motility were used in the study.

### Evaluation of *in vitro* sperm characters and freezing of semen

Neat semen samples were evaluated for volume, sperm cell concentration (hemocytometer method), mass activity, abnormal count (Rose Bengal staining method), individual motility, and functional membrane integrity (hypo-osmotic swelling test [HOST]) immediately after collection [4,5]. After the initial evaluation, semen ejaculates were diluted 1:5 with Tris-fructose-citric acid-egg yolk-glycerol buffer and equilibrated at refrigeration temperature for 3 h. Filling and sealing of straws were done manually in French Mini Straws during the equilibration period. After completion of equilibration, the semen straws were spread on plastic rack in a thermocol box (35 cm×17 cm×20 cm) 10 cm above the liquid nitrogen level in static liquid nitrogen vapor. After pre-freezing for 10 min in the vapor, the straws were then plunged directly into liquid nitrogen and stored at −196°C [4,5]. Semen samples were evaluated for *in vitro* characters such as individual motility, functional membrane integrity, and concentration of malondialdehyde (MDA) (MDA; thiobarbituric acid-trichloracetic acid method) after completion of the equilibration and post-freezethawing of sperm cells.

### Extraction and characterization of seminal proteins

The seminal plasma and sperm cells were separated immediately after collection by centrifugation (560 g for 10 min at 5°C). The sperm cells were washed with 2 ml of tris calcium chloride (TC) buffer (40 mM Tris, 2 mM CaCl_2_, and 0.01% sodium azide, pH 7.3) by centrifugation (560 g for 5 min at 5°C) to remove the leftover seminal plasma, if any. The sperm cells were resuspended with 1 ml of TC buffer containing protease inhibitor (1 mM phenylmethylsulfonyl fluoride) and washed thrice by centrifugation (560 g for 10 min at 5°C). The sperm pellet and the seminal plasma were stored at −20°C until extraction of protein [12]. Proteins in the seminal plasma were precipitated by ice-cold ethanol method [22]. Sperm proteins were extracted by Triton X detergent extraction method [23]. Sodium dodecyl sulfate-polyacrylamide gel electrophoresis (SDS-PAGE) was performed to characterize the proteins based on molecular weight [24]. The gels were stained with Coomassie Brilliant Blue and destained in a mixture of methanol (25%) and acetic acid (10%) in distilled water. The apparent molecular mass was determined using molecular weight markers and gel documentation and analysis system and the gels were stored in acetic acid (7%).

### Statistical analysis

Correlation between *in vitro* sperm characters and protein bands obtained was determined by Pearson’s correlation coefficient. Two-way ANOVA was applied to the effect of buck on *in vitro* sperm characters and different stages of semen preservation.

## Results

### *In vitro* sperm characters

Significant differences (p<0.01) in the *in vitro* sperm characters were observed among the bucks in the neat semen (Table-1), after equilibration period and post-freeze thawing (Table-2). The mean ± standard error of the mean (SEM) (range) of semen ejaculate volume 397.40±32.25 µl (260.20±6.66-490±16.33), sperm cell concentration 2517.50±69.63 million/ml (2329.60±10.43-3020.10±80.86), mass motility 3.20±0.21(2.70±0.15-4.60±0.16), individual motility 59.60±2.60% (49.90±2.38-72.40±1.06), functional membrane integrity 61.80±2.53% (51.50±1.20-73.20±3.38), and abnormal sperm count 4.80±0.20% (3.70±0.25-5.70±0.26) was recorded in the neat semen samples of Black Bengal bucks.

**Table-1:** *In vitro* sperm characters of neat semen samples in Black Bengal bucks.

Buck number	Volume (µl)	Concentration (millions/ml)	Mass motility	Individual motility (%)	Functional membrane integrity (%)	Abnormal count (%)
46	490±16.33^a^	2533.10±76.60^bc^	4.60±0.16^a^	72.40±1.06^a^	65.30±3.11^bc^	3.70±0.25^b^
48	485.20±36.55^a^	2594.10±31.48^ab^	3.40±0.26^b^	62.40±3.18^bc^	58.00±2.32^de^	5.00±0.26^ab^
51	485.20±56.29^a^	3020.10±80.86^a^	3.10±0.31^bc^	60.90±4.46^bcd^	73.20±3.38^a^	4.70±0.30^ab^
52	415.20±22.42^a^	2339.60±20.00^c^	2.90±0.23^bc^	49.90±3.57^e^	61.40±2.79^bcd^	4.90±0.31^ab^
53	425.20±13.43^a^	2417.40±36.54^c^	2.70±0.21^c^	49.90±2.38^e^	62.60±2.35^bcd^	4.40±0.40^ab^
55	455.20±8.97^a^	2406.60±65.10^c^	3.80±0.20^ab^	67.90±2.36^ab^	71.70±2.61^ab^	4.70±0.47^ab^
57	290.20±12.47^b^	2486.60±73.41^bc^	2.80±0.20^c^	63.40±1.83^ab^	60.70±3.00^cd^	4.80±0.33^ab^
59	270.20±8.16^b^	2329.60±10.43^c^	2.70±0.15^c^	54.40±1.38^de^	51.50±1.20^e^	5.70±0.16^a^
67	260.20±6.66^b^	2530.10±12.42^bc^	2.80±0.13^c^	55.40±1.17^cde^	51.80±0.92^e^	5.70±0.26^a^
Mean	397.40±32.25	2517.50±69.63	3.20±0.21	59.60±2.60	61.80±2.53	4.80±0.20

Data show all mean±SEM (n=10). Means in a column with different superscripts a, b, c, d, and e differ significantly at p<0.01. SEM=Standard error of the mean

**Table-2:** *In vitro* sperm characters after completion of equilibration and post-freeze–thaw in Black Bengal bucks.

Buck number	After completion of equilibration period			Post-freeze–thaw		
	
	Individual motility (%)	Functional membrane integrity (%)	Concentration of MDA (µ mol/ml)	Individual motility (%)	Functional membrane integrity (%)	Concentration of MDA (µ mol/ml)
46	56.50±1.42^a^	52.40±2.83^a^	0.28±0.08^a^	41.50±1.23^a^	42.30±1.60^a^	0.57±0.03^a^
48	45.50±1.74^b^	43.20±1.76^ab^	0.17±0.01^b^	30.00±1.67^b^	27.70±1.98^b^	0.32±0.03^a^
51	44.00±2.77^b^	48.90±2.59^ab^	0.31±0.06^ab^	26.50±1.83^b^	27.20±1.35^b^	0.28±0.04^a^
52	38.50±1.83^b^	39.10.±1.90^ab^	0.18±0.04^b^	28.50±1.30^b^	25.30±1.11^b^	0.38±0.03^a^
53	38.50±1.98^b^	46.60±2.26^b^	0.20±0.06^b^	33.00±2.00^ab^	30.80±1.57^ab^	0.45±0.04^b^
55	49.50±2.63^a^	51.10±2.98^a^	0.30±0.03^b^	40.50±1.74^a^	36.40±2.10^a^	0.63±0.06^a^
57	49.00±1.25^a^	44.30±1.45^b^	0.26±0.03^c^	34.50±1.17^ab^	27.10±0.65^b^	0.13±0.04^b^
59	45.56±1.74^b^	41.90±1.31^b^	0.50±0.05^c^	39.00±2.08^ab^	34.30±1.76^ab^	0.13±0.07^b^
67	46.00±1.80^b^	39.00±1.15^b^	0.56±0.61^c^	35.50±1.89^ab^	29.20±4.36^ab^	0.13±0.09^b^
Mean	45.89±1.85	45.00±1.64	0.31±0.04	34.30±3.78	31.10±1.84	0.33±0.06

Data show all mean±SEM (n=10). Means in a column with different superscripts a, b, and c differ significantly at p<0.01. SEM=Standard error of the mean, MDA=Malondialdehyde

The mean ± SEM (range) of individual motility 45.89±1.85% (38.50±1.83-56.50±1.42), functional membrane integrity 45.00±1.64% (39.00±1.15-52.40±2.83), and concentration of MDA 0.31±0.04 µmol/ml (0.20±0.06-0.56±0.61) was observed after equilibration period. The values were further deteriorated and significantly reduced (p<0.01) after freeze-thawing of sperm cells. Individual motility was significantly (p<0.01) reduced to 34.30±3.78% against 45.89±1.85% at equilibration, and functional integrity was significantly (p<0.01) reduced to 31.10±1.84% against 45.00±1.64% at equilibration. Significant difference among the bucks in their ability to withstand freezing injuries was noticed.

### Electrophoretic profile of seminal plasma proteins

A total of ten protein bands such as 180-136 kDa, 134-101 kDa, 75 kDa, 62-49 kDa, 48 kDa, 47-36 kDa, 35 kDa, 34-25 kDa, 20 kDa, and 17 kDa were observed in the SDS-PAGE of seminal plasma proteins (Table-3). Of these 10 bands, 75, 62-49, 20, and 17 kDa bands were present in all the nine bucks (100%), while the other proteins such as 180-136, 134-101, 48, 47-36, 35, and 34-25 kDa were present only 55.55%, 55.55%, 33.33%, 44.44%, 44.44%, and 44.44%, respectively, of the bucks screened.

**Table-3:** Electrophoretic profile of seminal plasma proteins of Black Bengal bucks assessed by SDS-PAGE.

Protein molecular weight (kDa)	Buck numbers									Overall presence of protein (n=9) n (%)

	46	48	51	52	53	55	57	59	67	
180-136	−	+	+	+	−	−	+	−	+	5 (55.55)
134-101	+	−	+	−	+	+	−	+	−	5 (55.55)
75	+	+	+	+	+	+	+	+	+	9 (100)
62-49	+	+	+	+	+	+	+	+	+	9 (100)
48	+	−	−	−	−	+	−	+	−	3 (33.33)
47-36	−	+	−	−	+	−	+	+	−	4 (44.44)
35	+	−	+	−	−	+	−	+	−	4 (44.44)
34-25	+	−	−	−	+	+	−	−	+	4 (44.44)
20	+	+	+	+	+	+	+	+	+	9 (100)
17	+	+	+	+	+	+	+	+	+	9 (100)

Figures in parentheses indicate percentage to total, − absence, + presence. SDS-PAGE=Sodium dodecyl sulfate-polyacrylamide gel electrophoresis

### Electrophoretic profile of sperm proteins

Electrophoretic profile of sperm proteins revealed the presence of nine bands starting from 17 to 134 kDa (Table-4). Proteins with molecular weight 75, 20, and 17 kDa were present in all the bucks screened (100%), while the other proteins such as 134-101, 100, 62-49, 63, 47-36, and 35 kDa were present only in 44.44%, 55.55%, 66.66%, 44.44%, 55.55%, and 33.33% of the bucks, respectively.

**Table-4:** Electrophoretic profile of sperm proteins of Black Bengal bucks assessed by SDS-PAGE.

Protein molecular weight (kDa)	Bucks numbers									Overall presence of protein (n=9) n (%)

	46	48	51	52	53	55	57	59	67	
134-101	+	−	−	−	−	+	+	+	−	4 (44.44)
100	+	+	+	+	+	−	+	−	+	5 (55.55)
75	+	+	+	+	+	+	+	+	+	9 (100)
62-49	+	+	+	+	+	−	−	−	+	6 (66.66)
63	−	−	−	+	+	+	+	+	−	4 (44.44)
47-36	+	−	+	−	−	+	+	+	−	5 (55.55)
35	+	−	+	−	−	−	+	−	−	3 (33.33)
20	+	+	+	+	+	+	+	+	+	9 (100)
17	+	+	+	+	+	+	+	+	+	9 (100)

Figures in parentheses indicate percentage to total, − absence, + presence. SDS-PAGE=Sodium dodecyl sulfate-polyacrylamide gel electrophoresis

### Correlation between seminal plasma proteins and *in vitro* sperm characters

Seminal plasma protein with molecular weight 180-136 kDa had shown a moderate positive correlation with sperm cell concentration (0.482) in neat semen (Table-5), while 134-101 kDa protein had shown a moderate positive correlation with functional membrane integrity (0.576) and 48 kDa shown a moderate positive correlation (0.454) with motility in post-equilibration period (Table-5). In the post-freeze–thaw evaluation, the 180-136 kDa protein showed a significant negative correlation with individual motility (−0.716) and functional membrane integrity (−0.724). Further, the protein band of 48 kDa showed a significant positive correlation with individual motility (0.649) and functional membrane integrity (0.664) and moderate positive correlation with MDA level (0.484). The protein band of 35 kDa showed a moderate positive correlation with individual motility (0.437) and functional membrane integrity (0.452), while the 33-25 kDa protein showed a moderate positive correlation with functional membrane integrity (0.422) and MDA level (0.568).

**Table-5:** Correlation between seminal plasma proteins and *in vitro* sperm characters.

Protein molecular weight (kDa)	Neat semen							After equilibration			Post-freeze thawing		
		
	Volume	Mass motility	Individual motility	Functional membrane integrity	Sperm concentration	MDA level	Abnormal count	Individual motility	Functional membrane integrity	MDA level	Individual motility	Functional membrane integrity	MDA level
180-136	0.004	−0.248	−0.16	0.077	0.482	−0.039	0.139	−0.294	−0.323	−0.085	−0.716	−0.724	−0.359
134-101	0.23	0.333	0.178	0.362	−0.039	0.325	−0.211	0.197	0.576	0.087	0.503	0.648	0.279
75	−0.207	0.266	0.146	−0.216	−0.25	−0.127	0.071	0.231	0.175	0.119	0.386	0.33	0.304
62-49	−0.07	−0.064	0.229	−0.149	0.035	0.154	−0.107	−0.148	−0.138	0.02	−0.015	0.025	0.12
48	−0.01	0.389	0.332	0.05	−0.374	0.251	−0.047	0.454	0.318	0.329	0.649	0.664	0.484
47-36	−0.209	−0.274	−0.282	−0.343	−0.282	−0.055	0.07	−0.209	−0.122	0.101	0.03	0.058	0.021
35	−0.069	0.309	0.172	0.115	−0.147	0.394	−0.143	0.327	0.244	0.216	0.437	0.452	0.306
34-25	0.164	0.246	0.152	0.184	−0.266	0.194	−0.248	0.134	0.345	0.165	0.369	0.422	0.568
20	0	0	0	0	0	0	0	0	0	0	0	0	0
17	0	0	0	0	0	0	0	0	0	0	0	0	0

MDA=Malondialdehyde

### Correlation between sperm proteins and *in vitro* sperm characters

The protein with molecular weight 134-101 kDa had shown a moderate negative correlation with ejaculate volume (−0.420), while 63 kDa protein showed a significant negative correlation with sperm cell concentration (−0.616) and moderate negative correlation with mass motility (−0.465) in the neat semen samples (Table-6). The 63 kDa protein showed a moderate negative correlation with individual motility (−0.414) in post-equilibration samples, and the 100 kDa protein had shown a moderate negative correlation with individual motility (−0.564) in post-freeze–thaw samples.

**Table-6:** Correlation between sperm proteins and *in vitro* sperm characters.

Protein molecular weight (kDa)	Neat semen							After equilibration			Post-freeze thawing		
		
	Volume	Mass motility	Individual motility	Functional membrane integrity	Sperm concentration	MDA level	Abnormal count	Individual motility	Functional membrane integrity	MDA level	Individual motility	Functional membrane integrity	MDA level
134-101	−0.42	−0.029	0.117	−0.262	−0.228	0.176	−0.132	0.257	−0.071	0.313	0.344	0.232	0.132
100	0.127	−0.195	−0.395	0.287	0.200	0.167	−0.021	−0.398	−0.0234	−0.144	−0.564	−0.374	−0.143
75	−0.009	−0.069	−0.158	−0.139	−0.224	0.164	−0.208	−0.15	−0.219	−0.25	−0.016	−0.066	0.009
62-49	−0.143	0.024	−0.363	−0.042	−0.008	0.08	−0.218	−0.329	−0.137	−0.312	−0.381	−0.173	−0.017
63	−0.337	−0.465	−0.372	−0.15	−0.616	0.005	−0.05	−0.414	−0.328	−0.149	0.082	−0.166	0.016
47-36	−0.223	0.095	−0.148	−0.115	0.021	0.259	−0.066	−0.178	0.108	0.155	−0.28	0.342	0.376
35	−0.276	0.058	0.218	0.214	0.23	0.16	−0.196	0.317	0.176	−0.199	0.084	0.132	−0.361
20	0	0	0	0	0	0	0	0	0	0	0	0	0
17	0	0	0	0	0	0	0	0	0	0	0	0	0

MDA=Malondialdehyde

## Discussion

AI in goat is gaining popularity, and it is essential to identify bucks with high fertility for donating semen. A prerequisite for selecting a male as semen donor is that it should have acceptable fertility after AI. Accurate evaluation of male fertility is important because it influences the reproductive potential of large herd. In recent years, several proteins had been linked in some way or another to fertilizing capacity of sperm. While most of these proteins were associated with the seminal plasma, some were identified in sperm. The role of seminal plasma proteins in the regulation of sperm function was highly complex and was manifested during different molecular events. Several studies provided direct evidence that proteins of seminal plasma were adsorbed to the surface of sperm and affected its function and properties [25]. This experiment was an attempt to characterize seminal proteins of Black Bengal buck semen and to find their correlation with *in vitro* sperm characters and semen freezability. In the present study, an average of 397.40 µl ejaculate volume, 2517.50 million/ml sperm cell concentration, 3.20 mass motility, 59.60% individual motility, 61.80% functional membrane integrity, and 4.80% abnormal sperm count was recorded in the neat semen of Black Bengal bucks. Semen ejaculates for the current experiment were collected during the period of November 2017-February 2018 which is winter season of the locality. However, in our previous observations, non-significant difference (p>0.05) was observed in the volume of ejaculate during different seasons; slightly larger volume of semen was obtained during the winter season (0.70±0.2 ml) and lower volume during rainy season (0.52±0.2 ml). Similarly, non-significant difference among the seasons was recorded for sperm motility also. Slightly higher motility was observed during winter (78.6±3.2) than rainy (76.6±3.4) and summer season (72.6±4.6). However, significantly (p<0.05) higher sperm cell concentrations were recorded during rainy season (3120×10^6^±180/ml) than during summer season (2440×10^6^±140/ml) in the Black Bengal buck semen ejaculates. Similar to the present study, 0.39±0.01 ml of ejaculate volume, 77.97±0.73% initial sperm motility, 3201.00±143.78×10^6^/ml sperm concentration, 83.02±0.65% live sperm, 7.66±0.73% sperm abnormality, 66.95±0.74% HOST-reacted sperm, and 93.34±0.51% intact acrosome were reported in the neat semen of Assam Hill goat (AHG) [22]. In another study, 0.77 ml ejaculate volume, 2.77×10^9^ ml^−1^ spermatozoa concentration, 3.19 mass activity, 68.57% individual motility, 15.5% dead cells, and 8.7% abnormal sperms were recorded in buck semen ejaculate [26].

The variations among the ejaculate characters in the different studies might be due to breed variations, frequency of semen collection, sexual preparation of bucks before collection, experience of the semen collector, management, and season of the year. It was observed that sperm motility and functional membrane integrity of the sperm cells were drastically reduced from their initial values after freeze-thawing of the semen. In concurrence to the observation, the loss of *in* *vitro* sperm characters was reported while freezing of Black Bengal buck semen in Triladyl and Tris-based diluents [27] and they had recorded 38.33% and 6.00% sperm motility in rethawed semen samples in Triladyl and Tris diluents, respectively. Significant (p<0.01) difference existed among the bucks in the sperm motility in fresh and frozen-thawed semen [27]. These findings indicate that there was progressive loss of *in* *vitro* sperm characters due to injury of cryopreservation. Cryopreservation induces detrimental effects in sperm cells, resulting in a loss of motility, membrane integrity, and fertilizing ability [28]. Under the best experimental conditions, about half the population of motile spermatozoa survive the freeze–thaw process. Buck sperm cells seem not well adapted to enduring cooling to low temperatures. There is a reduction of their post-thaw viability, as a consequence of accumulated cellular injuries that arise throughout the cryopreservation process [29].

Electrophoretic profile of seminal plasma Black Bengal buck semen revealed ten protein bands with molecular weight ranging from 17 to 180 kDa in this study. 16 protein bands of 14-97 kDa were reported in seminal plasma proteins of Anglo-Nubian goats [30], and 15 protein bands with molecular weight ranging from 15.13 kDa to 116.20 kDa were recorded in ram seminal plasma [31]. These variations might be due to the differences in the species, breed, methods of protein extraction and characterization. Electrophoretic profile of sperm proteins in this experiment revealed the presence of nine bands of 17-134 kDa, while the seminal plasma proteins had 10 bands. The 180-136 kDa protein band present in the seminal plasma of Bengal buck semen was not detected in the sperm proteins. SDS-PAGE of crossbred bulls’ semen (Jersey crossbred and Holstein Friesian crossbred) recorded 15 and 14 proteins, respectively, in the seminal plasma and sperm [17]. Sperm membrane proteins in AHG revealed 20 different protein bands with molecular weight ranging from 10 kDa to 75 kDa, and six bands such as 10, 14, 16, 49, 57, and 60 kDa were consistently present in all eight bucks [32].

Further, the protein with molecular weight 22, 30, and 38 kDa showed a frequency distribution of 87.50%, and 28, 45, and 47 kDa proteins had a frequency distribution of 75.00% in AHG. Seven protein bands with a molecular weight ranging from 29 to 200 kDa were detected in porcine semen [33]. The comparative sequence analysis revealed strong similarities between certain seminal plasma proteins identified in several species. The amino acid sequence of the ram seminal plasma proteins RSVP14 showed high homology with goat seminal plasma proteins GSP-14/15 kDa [34].

Seminal plasma proteins had positive/negative correlation(s) with *in vitro* sperm characters during cryopreservation of Black Bengal buck semen. 180-136 kDa protein shown a moderate positive correlation with sperm cell concentration (0.482) in neat semen, while 134-101 kDa protein shown a moderate positive correlation with functional membrane integrity (0.576) and 48 kDa protein shown a moderate positive correlation (0.454) with individual motility in post-equilibration period. In the post-freeze–thaw evaluation, 180-136 kDa protein showed a significant negative correlation with individual motility (−0.716) and functional membrane integrity (−0.724). Similarly, the sperm protein of 63 kDashowed a significant negative correlation with sperm cell concentration (−0.616) and moderate correlation with mass motility (−0.465) in the neat semen and moderate negative correlation with individual motility (−0.414) in post-equilibration samples. In buffalo bull semen, 24.5 kDa seminal plasma protein had a high correlation with sperm progressive motility in fresh and thawed semen, while 45 kDa groups were associated with unusual morphology in frozen-thawed semen; 55 kDa protein portions were connected with sperm viability of fresh semen [35]. Attempts have been made to analyze the roles of different protein fractions on the fertility of semen samples. Using two-dimensional gel electrophoresis, two seminal plasma proteins correlated with high fertility (26 and 55 kDa) and two proteins with low fertility (16 and 16 kDa) had been identified in bulls [36]. The 55-kDa fertility-associated protein has been identified as osteopontin [37], and the 26-kDa protein as lipocalin-type prostaglandin-D synthase [38]. HBP with a molecular weight of 28-31 kDa in the sperm membrane was named as fertility-associated antigen (FAA). Bull semen with the presence of FAA in sperm membranes had increased fertility by 9-40% points under natural service [38] and about 15% higher conception rate on AI. Spermatozoal FAA was shown to be a significant marker for fertility, even if bulls displayed similar behavioral serving capacities [39]. As like that of other species, identification of fertility-associated proteins in the buck semen will be of much useful for selection of breeding bucks for AI purpose.

## Conclusion

Variations among the bucks exist in their *in vitro* sperm characters, seminal plasma protein profiles, and ability to withstand freezing injury. Seminal proteins influence the *in vitro* sperm characters and could be used as a supplementary tool in addition to BSE for the selection of breeding bucks.

## Authors’ Contributions

MK designed the study. VCG collected the samples and performed the experiments. AM, MM, and SKD helped in running SDS-PAGE; MKG, SR, and RB helped in semen analysis, MK and AM analyzed the data. MK and VCG wrote the manuscript, and all authors read and approved the final manuscript.
